# The Nutraceutical Value of Carnitine and Its Use in Dietary Supplements

**DOI:** 10.3390/molecules25092127

**Published:** 2020-05-01

**Authors:** Alessandra Durazzo, Massimo Lucarini, Amirhossein Nazhand, Selma B. Souto, Amélia M. Silva, Patrícia Severino, Eliana B. Souto, Antonello Santini

**Affiliations:** 1 CREA-Research Centre for Food and Nutrition, Via Ardeatina 546, 00178 Rome, Italy; massimo.lucarini@crea.gov.it; 2 Department of Biotechnology, Sari Agriculture Science and Natural Resource University, 9th km of Farah Abad Road, Sari 48181 68984, Mazandaran, Iran; nazhand.ah@gmail.com; 3 Department of Endocrinology of Hospital São João, Alameda Prof. Hernâni Monteiro, 4200-319 Porto, Portugal; sbsouto.md@gmail.com; 4 Department of Biology and Environment, University of Trás-os-Montes e Alto Douro (UTAD), Quinta de Prados, P-5001-801 Vila Real, Portugal; amsilva@utad.pt; 5 Centre for Research and Technology of Agro-Environmental and Biological Sciences (CITAB), University of Trás-os-Montes e Alto Douro (UTAD), P-5001-801 Vila Real, Portugal; 6 Industrial Biotechnology Program, University of Tiradentes (UNIT), Av. Murilo Dantas 300, Aracaju 49032-490, Brazil; pattypharma@gmail.com; 7 Tiradentes Institute, 150 Mt Vernon St, Dorchester, MA 02125, USA; 8 Laboratory of Nanotechnology and Nanomedicine (LNMED), Institute of Technology and Research (ITP), Av. Murilo Dantas, 300, Aracaju 49010-390, Brazil; 9 Department of Pharmaceutical Technology, Faculty of Pharmacy, University of Coimbra, Pólo das Ciências da Saúde, Azinhaga de Santa Comba, 3000-548 Coimbra, Portugal; souto.eliana@gmail.com; 10 CEB—Centre of Biological Engineering, University of Minho, Campus de Gualtar, 4710-057 Braga, Portugal; 11 Department of Pharmacy, University of Napoli Federico II, Via. D. Montesano 49, 80131 Napoli, Italy

**Keywords:** carnitine, food supplements, nutraceuticals, in vitro studies, in animal studies, in humans studies

## Abstract

Carnitine can be considered a conditionally essential nutrient for its importance in human physiology. This paper provides an updated picture of the main features of carnitine outlining its interest and possible use. Particular attention has been addressed to its beneficial properties, exploiting carnitine’s properties and possible use by considering the main in vitro, in animal, and human studies. Moreover, the main aspects of carnitine-based dietary supplements have been indicated and defined with reference to their possible beneficial health properties.

## 1. Carnitine: An Overview of Its Main Features

**Figure 1:**
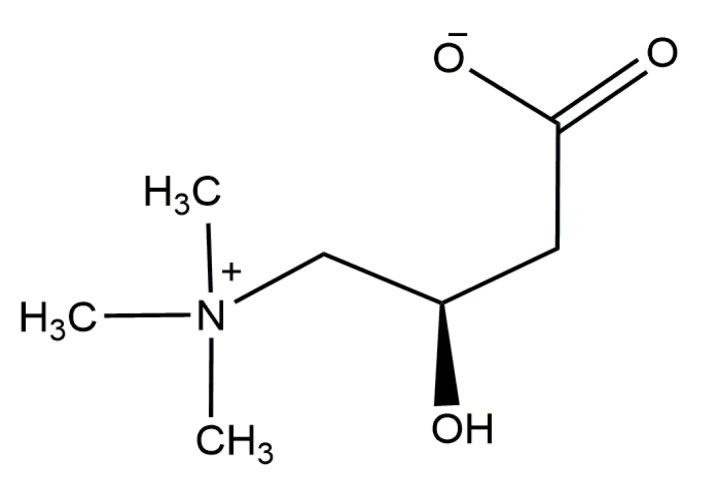
Chemical structure of L-carnitine.

Carnitine (n3-hydroxy-4-*N*,*N*,*N*-trimethylaminobutyrate; 3-hydroxy-4-(trimethylazaniumyl) butanoate (IUPAC name); β-hydroxy-γ-*N*-trimethylaminobutyric acid) is an amino acid derivative that exists as one of the d/l stereoisomers, l-carnitine being the biologically active isomer form of carnitine, naturally occurring in animals, while the d-carnitine is also active, but inhibits the effect of l-carnitine and is toxic. It is a naturally occurring endogenous metabolite which can be found in most mammals, it is a water-soluble molecule, and it has been reported to have many biological functions. Carnitine can be considered as a conditionally essential nutrient for its importance in human physiology [1]. The molecular structure of carnitine (C_7_H_15_NO_3_, MW = 161.2 g/mol, solubility >2500 g/L at a temperature of 20 °C; PubChem CID: 288) is shown in Figure 1. At a pH value of 3.8, it is a zwitterion, due to positively charged quaternary ammonium groups and carboxylate, and it dissolves easily in water at a temperature of 20 °C.

**Figure 2:**
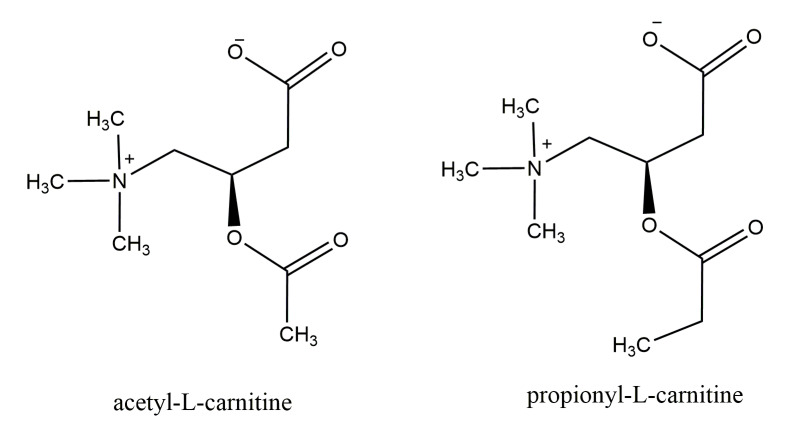
Chemical structure of acetyl-l-carnitine and propionyl-l-carnitine.

Carnitine is a vitamin-like substance (or “quasi-vitamins” [2]) and acts as a coenzyme, and its function is relevant, being necessary to deliver long-chain fatty acids (LCFAs) from the cytosol into the mitochondrial matrix. This can occur via carnitine palmitoyltransferase 1 (CPT1) in the outer mitochondrial membrane, thereby playing a role in energy supply to the body through the β-oxidation of LCFAs [3,4]. While defects in carnitine biosynthesis do not result in carnitine deficiency, severe plasma and intracellular carnitine depletion occurs due to defects in carnitine transport [5]. Carnitine homeostasis is maintained by diet and renal absorption (as only a small amount is obtained by endogenous biosynthesis). Renal absorption of carnitine occurs via cation transporter-2 (OCTN2; a high-affinity plasma-membrane sodium-dependent carnitine transporter), and by cation transporter-1 (OCTN1), with lower carnitine affinity than OCTN2. OCTN2 expression is not limited to kidney; it is found on many other cells (e.g., polarized intestinal cells, liver, heart, skeletal muscle, placenta, brains, and others) to guarantee carnitine absorption and distribution [5]. Defects in OCTN2, due to autosomal recessive mutations in the *SLC22A5* gene, result in carnitine deficiency, as a consequence of reduced carnitine transport and intracellular accumulation, increased urine excretion of carnitine and decreased serum levels of carnitine. Due to reduced intracellular levels of carnitine, defective fatty acid oxidation occurs, leading to glucose consumption instead of lipid consumption, even during fast, resulting in hypoglycemia; meanwhile, non-metabolized lipids (together with lipids released from adipose tissue) accumulate in tissues such as heart, skeletal muscle, and liver, resulting in myopathy and hepatic steatosis [6]. Therefore, researchers have proposed the measurement and detection of autosomal recessive carnitine deficiency in newborn screening programs [7,8,9,10]. Beyond its relevant role in fatty acid transport and oxidation, carnitine also acts as a free radical scavenger in different tissues, and also maintains cellular free coenzyme A levels [11,12]. The endogenous essential amino acids l-methionine and l-lysine are responsible for the formation of carnitine (known as a branched non-essential amino acid) in various tissues, such as brain, kidney, and predominantly liver. Meat, poultry, fish, and dairy foods, and, recently, dietary supplements supply 75% of carnitine [13]. The main animal source of carnitine is red meat (which contains up to 80 μg/100 g); it is present in moderate amounts in dairy products, and at a low-to-zero level in vegetables [14]. Endogenous production accounts for about 25%. Carnitine is easily absorbed from foodstuffs (up to 60–70%), but thermal treatments, e.g., cooking on open flames at high temperatures, can lower carnitine levels and consequently its bioavailability. Carnitine exists in two enantiomers, namely d-carnitine and l-carnitine, which are related to the presence of one chiral carbon: l-carnitine is the physiologically active form, as well as propionyl-l-carnitine and acetyl-l-carnitine, two derivatives which are also bioactive compounds. The structures of these derivatives are shown in Figure 2.

**Figure 3:**
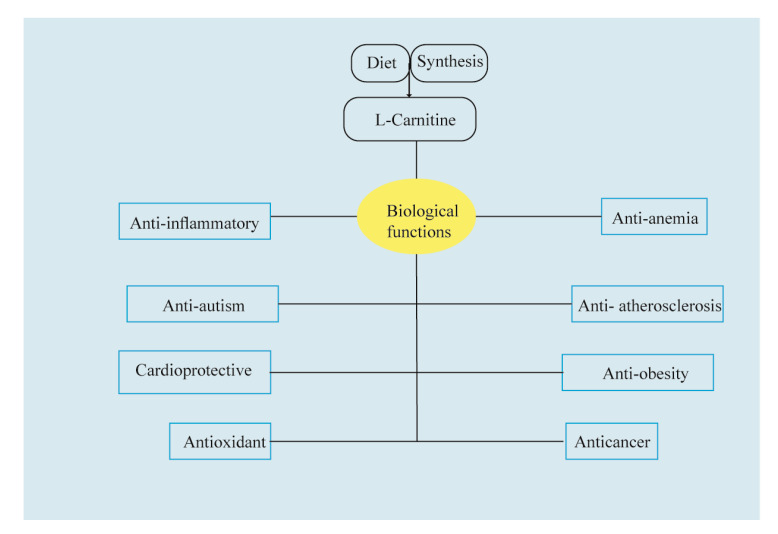
Overview of carnitine biological activities.

The biological functions of carnitine are summarized in Figure 3. Many health-beneficial actions of carnitine have been identified and reported, including suppression of apoptosis, correction of cytotoxicity by excessive acyl groups, and stabilization of the erythrocyte membrane [15] and anti-inflammatory and antioxidant properties [16]. It has also been reported that carnitine can improve insulin resistance [17], and it is useful in cardiovascular diseases [18,19] and cancer [20].

Moreover, it has been found by Hathcock et al. that carnitine can be considered a safe nutrient [21], with adverse outcomes typically limited to mild gastrointestinal discomfort when individuals ingest high dosages (>5 g/day). l-carnitine at a concentration of 2000 mg/day is considered to be unlikely to provoke unwanted side effects and is safe for human beings [21]. The administration of carnitine supplements has been documented for its safety and efficiency and reported in many studies [14,21].

## 2. An Updated Shot of Beneficial Properties: In Vitro and In Vivo Studies

Many in vitro studies [22,23,24,25,26,27,28,29], and in vivo studies of animals [30,31,32,33,34,35,36,37,38,39,40,41,42,43,44,45,46,47] and humans [48,49,50,51,52,53,54,55,56,57,58,59] have reported different beneficial functions for carnitine. In the following, the main activities observed are reported.

### 2.1. In Vitro Activity

Relevant in vitro studies on l-carnitine are reported in Table 1.

Carnitine showed in vitro anticancer activity by preventing colon cancer cell (Caco-2 cells) proliferation via a reduction in prostaglandin E2 synthesis and induction of colon cancer cell apoptosis [60]. In several prostate cancer cell lines, acetyl-l-carnitine acted as an anti-prostate cancer agent by inhibiting the production of chemokines CXCL12 and CCL2 as well as CXCR4 (chemokine ligand-receptor) and pro-inflammatory cytokines (IFN-γ and TNF-α) [61]. Huang et al. evaluated the in vitro anticancer role of carnitine in samples of HepG2 tumor-bearing mice, primary cultured thymocytes, human SMMC-7721, and hepatoma HepG2 cell lines. The main findings, following l-carnitine treatment, were prevention of cancer cell growth; inhibition of Histone Deacetylases HDAC I/II activities caused by l-carnitine binding to HDAC active sites; elevation of histone acetylation and acetylated lysine accumulation; and induction of p21^cip1^ gene, mRNA and protein expression in cancer cells [62].

Pre-treatment with acetyl-l-carnitine and l-carnitine exhibited neuroprotective activity for prevention of hypoxia-ischemia injury, via an increase in the activity levels of ATPase and superoxide dismutase (SOD) as well as a decrease in the level of malondialdehyde (MDA), oxygen-glucose deprivation (OGD)-induced cell death, injury and apoptosis [63]. The in vitro l-carnitine has been reported to reduce phenylalanine-induced DNA damage [64]. The administration of acetyl-l-carnitine (500 µM) exhibited a neuroprotective role by restoring synaptic plasticity and transmission [65]. In a study by Bavari et al. [66], the neuroprotective effect of l-carnitine (5 mM) controlled, within 18 h, caffeine cytotoxicity through the regulation of apoptosis-related caspase-3 activity, reducing the DNA fragmentation, inhibition of reactive oxygen species (ROS) generation, elevation of endogenous anti-oxidant defense systems, and the prevention of lipid oxidation. In vitro accumulated l-carnitine has been used to control DNA damage and oxidative damage in patients with mitochondrial fatty acid oxidation disorders [67].

l-carnitine has been also shown to manage fructose-induced hepatic steatosis in HepG2 cells, due to activation of antioxidant system, maintenance of mitochondrial homeostasis, and regulation of Nrf2 (nuclear factor erythroid 2–related factor 2) and SOD activity [68]. l-carnitine can increase antioxidant and mitochondrial functions in human osteoblast-like cells, via up-regulation of osteopontin, bone sialoprotein, transcription factor Sp7(Osterix), and RUNX2 genes; phosphorylation of AKT and ERK1/2; and enhanced phosphorylation of Ca^2+^/calmodulin-dependent protein kinase II [69]. In vitro post-oxidative stress glaucoma was controlled by carnitine through reducing pathologic optic-disk excavation, typical cell stress markers such as caspase 3 and ubiquitin, inducible nitric oxide synthase, and glial fibrillary acidic protein expression [70]. In a recent study, the lifespan of human mesenchymal stem cells obtained from aged participants was prolonged, by lengthening telomere and increasing the expression of the *hTERT* gene, following the use of l-carnitine (0.2 mM) for two days [71]. In an in vitro study, performed in blood leukocytes, by Rodrigues et al. [72], administration of L-carnitine (30 and 150 μM) prevented DNA damage induced by l-2-hydroxyglutaric and d-2-hydroxyglutaric in l-2-hydroxyglutaric-aciduria-affected patients. The effects of l-carnitine on different parameters of oxidative stress induced by menadione have been evaluated in myoblastic C2C12 cells: the results indicated a reduction in autophagy and ROS production [73]. In another recent study, human sperm morphology and sperm count were improved by 40 µg l-carnitine + coenzyme Q10 (CoQ10) treatment, while DNA fragmentation was reduced [74].

### 2.2. In-Animal Studies

Table 2 presents an updated picture of studies based on l-carnitine which have been conducted in animals.

Taking the anti-histamine drugs cetirizine hydrochloride and chlorpheniramine maleate combined with l-carnitine showed a hepatoprotective effect in animal models, via a reduction in oxidative stress and an improvement in liver function due to the elevation of serum albumin levels and a reduction in serum alkaline phosphatase (ALP), aspartate transferase (AST), and alanine transferase (ALT) levels; this treatment also enhanced hepatic glutathione (GSH) levels and reduced hepatic MDA compared with a control [75]. Ahmed et al. used 50 mg/kg/day of atorvastatin in rats for induction of hepatoxicity, and then utilized 300 mg/kg/day of oral l-carnitine and 500 IU/kg/day of oral vitamin D3. Their results showed a decrease in serum levels of creatine kinase, aspartate aminotransferase, and alanine aminotransferase. Furthermore, histological examinations revealed protection of muscle and liver tissues against the toxic effects of atorvastatin [76]. The use of nicotinamide riboside plus l-carnitine in high-fat-diet-treated mice with non-alcoholic fatty liver disease induced anti-obesogenic hepatoprotective activity due to regulation of INSR, PPARGC1B, SREBF, SCAP, and ACOX, as well as reduction of hepatic steatosis, fat mass, and obesity [77]. Administration of 200 mg/kg/day of coenzyme Q10 plus 50 mg/kg/day of l-carnitine in CCl4 hepatoxicity-induced rats exhibited the prophylactic effect observed [78]. In a recent study, l-carnitine showed a free radical scavenger effect in ethanol-intoxicated rats through the inhibition of hepatocyte function modification [79]. Induction of renal and pancreatic injuries by cyclosporine (15 mg/kg/day) in rats was treated by l-carnitine (50 or 200 mg/kg/day) for a month, the result of which was inhibition of LC3-II and caspase-3 expression, suppression of 8-OHdG and TGF-β1 expression, improved inflammation and renal function, decreased HbA1c and plasma glucose levels, and elevated plasma insulin level [80]. In another study, the use of l-carnitine prevented non-alcoholic steatohepatitis in mice by blocking inflammatory cytokines, preventing hepatic oxidative stress markers and elevating hepatic gene expression [81].

The administration of acetyl-l-carnitine prevented atherosclerosis onset in Wistar rats by blocking the expression of oxidative-stress-related genes, controlling inflammation parameters, and regulating blood lipids, as well as displaying myocardial protection and acting against atherosclerotic cardiovascular disease, by reducing mRNA levels, iNOS, IL-1b, TNF-a and CPR protein in the hearts and aortas of rats with induced atherosclerosis [82]. Blanca et al. found that sunitinib produced cardiac toxicity via the involvement of fibrotic and inflammation processes mediated by transcription factor NF-kB, and they also reported that l-carnitine showed a protective effect against secondary fibrotic process, cardiac inflammation, and arterial hypertension induced with sunitinib in Wistar rats [83]. Co-administration of 300 mg/kg of l-carnitine plus 10 mg/kg of olmesartan could control doxorubicin-induced (20 mg/kg) cardiotoxicity in rats, through elevation of cardiac levels of glutathione and superoxide dismutase and reduction of cardiac levels of malondialdehyde, transforming growth factor Beta, NF-kB, myeloperoxidase, Interleukin IL-1β, intercellular adhesion molecules-1, tumor necrosis factor-alpha and caspase-3 [84]. The supplementation of propionyl-l-carnitine in a hamster cheek pouch with ischemia–reperfusion injury prevented microvascular modifications through a reduction in E-selectin expression, resulting in permeability enhancement and poor leukocyte adhesion [85]. In one study, l-carnitine showed an antihypertensive function in rats with heart failure with preserved ejection fractions through an increasing the prostacyclin synthesis and the expression of fatty acid desaturase, respectively [86]. The use of l-carnitine (400 mg/kg/day) had a health-promoting effect on hypertension-associated cardiac fibrosis in rats through the down-regulation of CTGF, TGF-β1 and NOX2/4, and a reduction in cardiac fibrosis [87].

The use of l-carnitine plus selenium for a month to treat cadmium-induced damage in male mice during could reduce DNA damage and histopathological abnormalities and increase the activity of antioxidant enzymes [88]. The treatment of rats with l-carnitine (50 mg/kg/day) for 7 months exhibited antioxidant properties by regulating Bax and Bcl-2, dropping caspase-3 activity, elevating total antioxidant activity, and scavenging oxygen free radicals [89]. Others reported that the administration of L-carnitine (500 mg/kg) showed antioxidant and protective effects on testicular ischemia-reperfusion damage in rats [90]. In a study by Boyacioglu et al., the use of L-carnitine in rats controlled contrast-induced nephropathy through a preventative function [91].

In a recent study, adult male rats received busulfan plus l-carnitine/arginine, and, as a result, showed reduced busulfan cytotoxicity, conserved cell energy, reduced oxidative stress, and better semen parameters [92]. Masoumi-Ardakani et al. [93] administered 300 mg/kg/day of l-carnitine to 48 male rats with Streptozotocin-induced diabetes for 35 days and observed an increase in pancreatic and serum levels of glutathione peroxidase, superoxide dismutase, and total antioxidant status. Others reported that bone microstructural features were improved and bone resorption was slowed following the administration of l-carnitine in aging ovariectomized rats, due to the reduction in bone turnover [94]. Evaluating the effect of hyperbaric oxygen on lipid metabolism dysfunction in high fat diet-fed mice showed an increase in the expression of PPARα, skeletal muscle and circulation levels of l-carnitine [95]. In one study, 300 mg/kg/day of acetyl-l-carnitine for 28 days in rats attenuated OP-induced haemotoxicity [96].

### 2.3. In Human Studies

An up-to-date picture of studies conducted in humans is presented in Table 3.

The findings of a recent systematic review and meta-analysis of randomized clinical trials regarding l-carnitine supplementation showed an amelioration of muscle soreness and an improvement in muscle damage biomarkers, due to a decrease in lactate dehydrogenase, myoglobin and creatine kinase [97]. In a study by Chae et al. [98], daily administration of two or three 500-mg/ l-carnitine tablets reduced imatinib-induced muscle cramps in gastrointestinal stromal tumor (GIST) patients. A 750 mg/day dose of l-carnitine for 8 weeks in female patients with knee osteoarthritis reduced the pain intensity and serum inflammatory mediators such as matrix metalloproteinases-1 and Interleukine-1b [99]. In a recent study, supplementation of l-carnitine (750 mg/day) for 8 weeks in patients with osteoarthritis caused knee pain improvement and reduced serum inflammatory markers (namely MMP-1, and IL-1β) [99].

Kazemian et al. [100] reported a neuroprotective function in 100 patients with ischemic cerebrovascular injury, following the use of fat emulsion and l-carnitine, which decreased S100B biomarker levels. Reportedly, patients with painful peripheral neuropathy have been treated by the administration of acetyl-l-carnitine [101].

Abolfathi et al. [102] conducted a clinical trial to evaluate the effect of carnitine in patients with nonalcoholic fatty liver disorder and found a decrease in homeostasis model assessment of insulin resistance, triglycerides, alanine aminotransferase, and aspartate aminotransferase. In a systematic review and meta-analysis by Thiagarajan et al. [103], the effect of dietary l-carnitine supplementation was reviewed quantitatively and qualitatively in randomized trials (sample size of 338 individuals) from Iran, South Korea, and Italy. Decreases in Homeostatic Model Assessment for Insulin Resistance (HOMA-IR), liver fat, and serum alanine aminotransferase have also been reported [103]. Oral l-carnitine showed a hepatoprotective effect due to a decrease in the levels of gamma-glutamyltransferase, aspartate aminotransferase, and alanine aminotransferase [104].

l-carnitine supplementation in elderly hemodialyzed patients with end-stage kidney disorder increased amino acid metabolism, fatty acid metabolism, blood acy l-carnitine levels, and energy production in skeletal and heart muscles [105]. Others found 50 mg/kg l-carnitine for 6 to 10 months in children treated with hemodialysis pediatric at 18 years of age by reduction of parathyroid hormone level, which led to maintained bone density and decreased bone resorption [106]. In one study, in pediatric peritoneal dialysis, patients observed reduction of apolipoprotein B levels with 50 mg/kg per day of l-carnitine supplementation for a month [107]. Sheikhi et al. [108] found no change in Apo AI and SB100 levels following use of l-carnitine. One study outlined the great risk of carnitine deficiency: carnitine insufficiency and the prevalence of carnitine deficiency were estimated at 73.5%, 82.3% and 8.8% among dialyzed patients, respectively [109]. l-carnitine displayed a cardioprotective function in women after six months, increasing d-loop methylation in platelets and reducing the low-density lipoprotein cholesterol level and trimethylamine-*N*-oxide level [110]. Bavbek et al. [111] reported that the use of carnitine (20 mg/kg) three times a week for six months in chronic hemodialyzed patients could increase total and free carnitine levels by improving forced expiratory volume in one second and forced vital capacity, leading to management of respiratory dysfunction.

Patients with phenylketonuria receiving l-carnitine (98 mg/day) combined with selenium (31.5 micrograms/day) for six months showed the restoration of GSH-Px activity, and the improvement of protein and lipid oxidative damage [112]. Chromium picolinate (at a dose of 200 µg/day) combined with carnitine (1000 µg/day) in women with polycystic ovary syndrome for 12 weeks showed a significant improvement of stress, depression, and anxiety [113].

Administration of l-carnitine in patients with maple syrup urine disease prevented DNA damage induced by alloisoleucine, branched-chain α-keto-acids and branched-chain amino acids [114]. In a study by Cruciani et al. [115], patients affected by cancer with carnitine deficiency-induced fatigue received l-carnitine (3000 mg/day) for a week, and showed a significant improvement, enhancing total and free carnitine without any toxicity or complications. Use of l-carnitine (4 g/day) for three months in patients with advanced pancreatic cancer improved quality of life and nutritional status [116].

In a study by Shirali et al. [117], male teen soccer players received carnitine (2 g/day) combined with caffeine (6 mg/kg/day) and showed a reduction in body weight and body fat percentage by increasing lipolysis. Use of l-carnitine (250 mg) for 12 weeks in patients with polycystic ovary syndrome reduced hip and waist circumference, body mass index, and weight [118]. In a study conducted in Libia by Ibrahim et al., 1000-mg l-carnitine supplementation twice a day for three months in type 2 diabetic patients with dyslipidemia reduced triglyceride levels, but did not change low-density lipoprotein-cholesterol, high-density cholesterol lipoprotein or total cholesterol levels [119].

Moradi et al. [120] improved idiopathic male infertility through the administration of clomiphene citrate and carnitine, so that their patients receiving carnitine (25 mg/day) showed an increase in semen volume and those receiving clomiphene citrate (2 g/day) showed an improvement in morphology and motility. In another study, patients with chronic hepatitis C virus infection who received l-carnitine (2 g twice a day) combined with ribavirin (800 mg/day) for 12 months showed an improvement in sustained virological response and modulation of thrombocytopoiesis, leucopoiesis and erythropoiesis [121]. The use of l-carnitine (50 mg/kg/day) in patients with maple syrup urine disease for two months diminished DNA damage index [122].

## 3. Carnitine-Based Dietary Supplements

Nowadays, food supplements field is certainly varied and growing: a great range of new products are launched on the market every year. This is reflected in a new reorganization of drugs leading to changes in dietary supplement regulations [123,124,125,126,127,128,129,130,131,132,133,134,135,136]. Dietary supplements are made by mixing biologically active substances intended for consumption with food or as an addition to food products, with the purpose of optimizing metabolic processes and human body functions. Dietary supplements include, mainly, micronutrients, e.g., vitamins, trace elements, amino acids and enzymes, but also proteins, probiotics and oils, which can provide antioxidant, detoxifying, immunomodulatory and adaptogenic effects, etc.

Virji, in a 2017 study [137], remarked on the potential benefits of l-carnitine as dietary supplement. There is a growing body of outcomes data that demonstrates the beneficial effects of l-carnitine in the treatment of coronary artery disease, metabolic syndrome, and obesity. Odle et al., in 2014, reported how the l-isomer can be synthesized, and, consequently, high-purity dietary supplements are commercially available and are generally recognized as safe [14].

### 3.1. Monitoring l-Carnitine in Dietary Supplements

In the context of dietary supplements, detection of physiologically active components represents a difficult task and requires the use of modern highly informative research methods. Some studies describing this methodological approach to l-carnitine in dietary supplements are reported in the following. De Andrés et al. [138] proposed achiral liquid chromatography with circular dichroism detection for the determination of carnitine enantiomers in dietary supplements and pharmaceutical formulations [138]. Sánchez-Hernández et al. [139] developed a method for simultaneous and simple unequivocal identification and determination of carnitine enantiomers in dietary food supplements by capillary electrophoresis–electrospray ionization–tandem mass spectrometry [139]. Isaguirre et al. [140] proposed a new flow injection method for quality control of dietary supplements containing l-carnitine using an extraction mediated by sodium taurodeoxycholate coacervate coupled to molecular fluorescence [140]. A recent work of Voitiuk et al. [141] proposed a simple, rapid and selective method for determining ascorbic acid and l-carnitine l-tartrate in a multicomponent dietary supplement, produced in the form of sachets, using HPLC with spectrophotometric detection [141]. Ellingson et al. used LC-MS/MS-based analysis to measure total and free carnitine levels, so that acid-assisted microwave hydrolysis and water extraction were employed to analyze total and free analysis respectively. Their results showed overall RSD with intermediate precision of 3.1% and 3.3% and an overall repeatability of 2.7% and 2.9% for total and free carnitine levels, respectively [142]. Johnson et al. applied LC-MS/MS analysis to measure the plasma levels of total and free carnitine. Acetonitrile 0.3% formic acid was used to extract the total carnitine and to avoid the time-consuming step of salt elimination, and acid hydrolysis was utilized instead of base hydrolysis to quantify the total carnitine level [143]. The LOQ and LOD values were 2.54 and 1.79 μmol/L for the total carnitine and 1.36 and 0.87 μmol/L for the free carnitine. The varying analytical techniques used seem to indicate that the monitoring can be achieved by using different techniques but also that more in-depth studies are needed to identify a unique method of analysis which can guarantee efficient monitoring of the amount of the active components present in a food supplement.

### 3.2. A Shot of Dietary Supplement Label Databases

Considering the relevance of dietary supplements in the evaluation of total dietary intake, remarked on during the National Health and Nutrition Examination Survey (NHANES), a dietary supplement label database [144,145] was launched in 2013 by the Academy of Nutrition and Dietetics in the United States: this contains label information (brand name, ingredients, amount per serving, and manufacturer contact information) on more than 71,000 dietary supplements present and consumed in the U.S. marketplace [144,146,147,148]. The dietary supplement label database (DSLD) can be used to track changes in product composition and capture new products entering the market, representing a useful tool for consumers, professionals, and researchers, useful for multiple applications [144,147]. For example, by searching in DSLD [146] by product/brand name, and typing “carnitine” as a keyword, research has identified 434 products.

Recently, at European level, information on the compositions reported on labels of selected dietary supplements has been collected and updated for the development of a DSLD according to products’ availability on the Italian market, also including items consumed in the last Italian Dietary Survey [149,150]: a total of 558 products have been entered into the database as of December 2019. This aims to give a uniform image and representation of the major classes of food supplements consumed in Italy. It is important to underline that, for each item, a code was assigned following the food classification system FoodEx2 developed by EFSA [151], to allow standardization and harmonization of the data among different countries.

In particular, DSLD in Italy reported the codes for seven carnitine-based products, classifying, with a base term [A03SC] Carnitine or creatine-based supplements for sports people. Carnitine is also present as an ingredient in Mixed supplements/formulations [A03TC] as well as in Micronutrients supplement for sports people [A03SB].

## 4. Conclusions and Future Remarks

This perspective paper offers an updated overview of the nutraceutical value of carnitine and its use in dietary supplements. The beneficial health effects observed are many.

Nonetheless, the use of carnitine as dietary supplement should be regulated to avoid overdosage. Some studies show a possible onset of side effects related to carnitine supplements. These unwanted effects include sporadic vomiting and diarrhea, as observed in children with autism spectrum disorder when administered high doses of carnitine, e.g., 400 mg/kg/day [152]. The recent review by Malaguarnera and Cauli [153], summarizing the effects of l-carnitine in patients with autism spectrum disorders, reported that doses of about 50–100 mg/kg/day are generally well tolerated, whereas side effects observed with a dose of 200 mg/kg/day resulted in gastro-intestinal symptoms and a strong, unpleasant skin odor. On the other hand, a recent meta-analysis published by Asadi et al. [154] showed that l-carnitine supplementation could be effective in maintaining lipid profile levels, particularly in doses higher than 1500 mg/day, even if more RCTs with large sample sizes, focusing on gut microbiome profiles and dietary patterns, are needed. The limited number of clinical trials evaluating the effects of carnitine the human health seems to emphasize the need for and importance of further research in this field.

## Figures and Tables

**Table 1:** An up-to-date picture of in vitro studies of l-carnitine.

Condition	Activity	Effect	References
*In vitro*	Anticancer effects	Reduced the levels of methylmalonicacidemia and Propionic acidemia in peripheral leukocytes.	[22]
*In vitro*	Antioxidant effects	l-carnitine could elevate in vitro human sperm motility.	[23]
*In vitro*	Antioxidant effects	Inhibited acrylamide-induced genotoxicity in human lymphocytes through the improvement of oxidative stress.	[24]
*In vitro*	Antioxidant effects	Inhibited ROS production and reduced antioxidant activity.	[25]
*In vitro*	Anti-aging effect	Decreased epigenetic modification of hTERT gene promoter and the numbers of senescent cells, and increased activity of telomerase.	[26]
*In vitro*	Hepatoprotective effect	Inhibited the inflammatory mediator iNOS through the suppression of NF-kB activity in IL-1β-stimulated hepatocytes.	[27]
*In vitro*	Anti-angiogenic effect	Suppressed the activation of ICAM-1 and NF-kB and down-regulated the activation of FAK, CXCR4, CXCL12, VEGFR2 and VEGF pathways.	[28]
*In vitro*	Neuroprotective effect	Inhibited methamphetamine-induced activation of MMP-9	[29]

**Table 2:** An updated picture of studies based on l-carnitine conducted inanimal models.

Condition	Activity	Effect	References
In-animal model	Antioxidant effects	Symptom improvement observed by inducing potential function of the CNS and short-term plasticity.	[30]
In-animal model	Antioxidant effects	Impeded age-related mitochondrial dysfunction by reducing oxidative stress, age-related alterations of mitochondrial dynamics and biogenesis, and activation of PGC-1α/β coactivators.	[31]
In-animal model	Anti-diabetic effects	An improvement of glucose metabolism in mice with insulin resistant	[32]
In-animal model	Anti-diabetic effects	Reduction in the serum levels of adiponectin.	[33]
In-animal model	Anti-inflammatory and anti-oxidant effects	Managed histological and inflammation damage, apoptosis, mitochondrial dysfunction and arsenic-induced hepatotoxicity.	[34]
In-animal model	Antioxidant effect	Upregulation of *nrf2* expression and elevation of GSH and TAC levels.	[35]
In-animal model	Cardioprotective effect	Controlled the cardiac toxicity induced by 75- and 150-mg/Kg BW aspartme.	[36]
In-animal model	Anti-obesity effect	Reduction in elevated plasma lipids in obese Zucker rats.	[37]
In-animal model	Immunostimulatory and radioprotective role	Reduced sperm abnormalities, modified severe tubular degeneration and increased serum testosterone levels.	[38]
In-animal model	Enhanced exercise endurance	Reduced body fat, increased maximum running time, and elevated mitochondrial biogenesis, oxidative metabolism and fatty acid adsorption.	[39]
In-animal model	Cardioprotective effect	Inhibited 6-Gy γ-radiation-induced toxicity.	[40]
In-animal model	Antioxidant effect	Prevented NaAsO2-induced oxidative damage in rat.	[41]
In-animal model	Treatment of muscle atrophy	Prevented muscle atrophy by inhibiting the ubiquitin proteasome pathway.	[42]
In-animal model	Anti-atherosclerosis effect	Prevented the production of trimethylamine N-oxide.	[43]
In-animal model	Antioxidant effect	Decreased the oxidative stress at least in the heart of oophorectomized rats.	[44]
In-animal model	Antioxidant effect	Decreased acrylamide-toxicity in spleen and thymus tissues in mice.	[45]
In-animal model	Antioxidant effect	l-carnitine (200 mg/kg BW) for 11 weeks prevented dimethoate toxicity in rats.	[46]
In-animal model	Antioxidant effect	Reduction in PCC (protein oxidation marker), TBARS (lipid peroxidation marker), caspase-3, DNA fragmentation, cyclobutane pyrimidine dimers, 8-oxo-2′-deoxyguanosine (8-oxo-dG) as well as proinflammatory cytokines IL-1β, IL-6, and TNF-α downregulation, upregulation of PCNA (DNA repair proliferating cell nuclear antigen) protein, removed c-Fos and oxidative stress-sensitive signaling protein p38.	[47]

**Table 3:** An up-to-date picture of in vivo studies of l-carnitine.

Condition	Activity	Administration	Effect	References
Clinical trial	Cardioprotective effect	Daily oral l-carnitine (50 mg/kg) in patients with ischemic heart failure for 10 days	Enhancement of cardiac efficiency, restoration of cardiac energy metabolism, and elimination of toxic mitochondrial products.	[48]
Clinical trial	Cardioprotective effect	l-carnitine supplementation at the concentration of 2 g/day for 8 weeks in patients with Pemphigus vulgaris	Reduced serum levels of cystatin C, BMP4 and OPN as well as increased serum levels of carnitine.	[49]
Clinical trial	Anti-inflammatory effects	Administration of carnitine (250 mg/day) in females with polycystic ovary syndrome for 12 weeks	Decreased carotid intima-media thickness (CIMT) and plasma nitric oxide.	[50]
Clinical trial	Antioxidant effect	l-carnitine supplementation at the concentrations of 10 mM and 30 mM for 55 days	Elevated sulfhydryls and ascorbic acid uptake, preserved glutathione level, enhanced sulfhydryls and ascorbic acid levels, preserved lipid peroxidation, haemolysis and haemoglobin, and modulated antioxidants.	[51]
Clinical trial	Antioxidant effects	Administration of l-carnitine (100 mg/kg day) in patients with glutaric acidemia type I for 2 month	Prevented oxidative damage and increased the removal of toxic metabolites in patients with type I glutaric aciduria.	[53]
Clinical trial	Embryonic development effect	Administration of l-carnitine (1000 mg/day) for 82 days	An improvement of oocyte developmental competence in patients with *in-vitro* fertilization-embryo transfer.	[55]
Clinical trial	Anti-anemia effect	The administration of l-carnitine (20 mg/kg/day) for three months in dialysis children	A restoration and normalized circulation of plasma free carnitine (FC) levels	[56]
Clinical trial	Anti-autism effect	Administration of l-carnitine (50 mg/kg/day bodyweight) for three months	An improvement of autism symptoms based on autism treatment evaluation checklist (ATEC) scores, modified clinical global impression (CGI), and childhood autism rating scale (CARS)	[57]
Clinical trial	Anti-autism effect	Administration of l-carnitine (100 mg/kg/day body weight) in children	An enhancement of total and free carnitine levels, a reduction of autism severity and an improvement of autistic behavior	[58]
Clinical trial	Anti-autism effect	Administration of l-carnitine (200 mg/kg/day) in male subjects aged 5 years for 4.5 months	A gradual improvement of autism symptoms	[59]
